# Bee-derived antibacterial peptide, defensin-1, promotes wound re-epithelialisation *in vitro* and *in vivo*

**DOI:** 10.1038/s41598-017-07494-0

**Published:** 2017-08-04

**Authors:** Marcela Bucekova, Martin Sojka, Ivana Valachova, Simona Martinotti, Elia Ranzato, Zoltan Szep, Viktor Majtan, Jaroslav Klaudiny, Juraj Majtan

**Affiliations:** 1 Laboratory of Apidology and Apitherapy, Institute of Molecular Biology, Slovak Academy of Sciences, Dubravska cesta 21, 845 51 Bratislava, Slovakia; 2 Institute of Zoology, Slovak Academy of Sciences, Dubravska cesta 9, 845 06 Bratislava, Slovakia; 3 Department of Microbiology, Faculty of Medicine, Slovak Medical University, Limbova 12, 833 03 Bratislava, Slovakia; 4 Faculty of Natural Sciences, Comenius University, Ilkovicova 6, 842 15 Bratislava, Slovakia; 5 DiSIT-Dipartimento di Scienze e Innovazione Tecnologica, University of Piemonte Orientale, Viale Teresa Michel 11, Alessandria, 15121 Italy; 6 DiSIT-Dipartimento di Scienze e Innovazione Tecnologica, University of Piemonte Orientale, piazza Sant’Eusebio 5, Vercelli, 13100 Italy; 7 Cytopahtos Laboratory, Kutuzovova 23, 831 03 Bratislava, Slovakia; 8 Institute of Chemistry, Slovak Academy of Sciences, Dubravska cesta 9, 845 38 Bratislava, Slovakia

## Abstract

Royal jelly (RJ) has successfully been used as a remedy in wound healing. RJ has multiple effects, including antibacterial, anti-inflammatory and immunomodulatory activities, in various cell types. However, no component(s) (other than antibacterial) have been identified in RJ-accelerated wound healing. In this study, we demonstrate that keratinocytes are responsible for the elevated production of matrix metalloproteinase-9 (MMP-9) after incubation with a water extract of RJ. Furthermore, the keratinocyte migration and wound closure rates were significantly increased in the presence of RJ extract. MMP-9 production was reduced significantly following proteinase K treatment but remained stable after heat treatment, indicating that active component(s) have a proteinous character. To identify the component responsible for inducing MMP-9 production, RJ extract was fractionated using C18 RP-HPLC. In fractions exhibiting stimulatory activity, we immunochemically detected the bee-derived antibacterial peptide, defensin-1. Defensin-1 was cloned, and recombinant peptide was produced in a baculoviral expression system. Defensin-1 stimulated MMP-9 secretion from keratinocytes and increased keratinocyte migration and wound closure *in vitro*. In addition, defensin-1 promoted re-epithelisation and wound closure in uninfected excision wounds. These data indisputably demonstrate that defensin-1, a regular but concentration variable factor found in honey and RJ, contributes to cutaneous wound closure by enhancing keratinocyte migration and MMP-9 secretion.

## Introduction

Keratinocytes, a major cellular component of the epidermis, are responsible for restoring the epidermis after injury through a process termed epithelialisation. The migration, proliferation, and differentiation of fibroblasts and keratinocytes, as well as interactions between these cells are critical for effective re-epithelialisation and wound healing. Immature keratinocytes produce matrix metalloproteinases (MMPs), including MMP-9 and MMP-2, and plasmin, which enables their dissociation from the basement membrane and facilitates their migration. MMP-9 (gelatinase B) is a zinc-dependent endopeptidase that is involved in the proteolytic degradation of extracellular matrix proteins, such as type III and IV collagens and elastin. MMP-9 plays an important role in normal wound healing, particularly related to extracellular matrix (ECM) remodelling and re-epithelialisation. Wound healing is impaired when MMP-9 is inhibited^1, 2^.

Historically, honeybee products, such as honey and royal jelly (RJ), have been used to treat a broad spectrum of injuries. RJ is part of the diet of honeybee larvae and is secreted from the hypopharyngeal and mandibular glands of worker honey bees^3^. RJ has been used since ancient times to facilitate wound healing. RJ acts as an antimicrobial^4–8^ and antioxidative agent^9^ and as an immunomodulator with anti-inflammatory properties^10, 11^.

The topical application of RJ to treat human diabetic foot ulcers^12–14^ provides compelling evidence that RJ can accelerate wound healing. Furthermore, RJ promotes wound healing in an animal model of uninfected wound^15^. However, the mechanisms of action, other than antibacterial effects, associated with the effects of RJ in wound healing remain enigmatic. In fact, relatively few studies have investigated the influence of RJ and/or its components on human skin cells (epidermal keratinocytes and dermal fibroblasts) that are involved in wound healing^16–20^. These studies revealed that RJ enhances the migration of dermal fibroblasts, alters the levels of various lipids involved in the wound healing process^17^, and increases the production of type I pro-collagen and transforming growth factor β (TGF-β) by fibroblasts^16, 19^. In addition, 10-hydroxy-2-decenoic acid (10-HDA), a specific fatty acid found in RJ, induces involucrin, transglutaminase-1 and filaggrin protein production by human keratinocytes^20^.

In this study, we identified and characterised a component of RJ that induces MMP-9 secretion and keratinocyte migration *in vitro*. This component also improves wound closure and re-epithelialisation in rats and thus promotes wound healing *in vivo*.

## Results

### RJ induces MMP-9 production in HEK and HaCaT cells

We initially measured the cytotoxicity of WRJE on HaCaT cells using the Alamar blue assay. WRJE did not reduce cell viability at the tested concentrations, which ranged from 25 to 1000 µg/ml (Supplementary Fig. S1). Similarly, no cytotoxic effect was noted in HEK cells at concentrations up to 1000 µg/ml.

**Figure 1:**
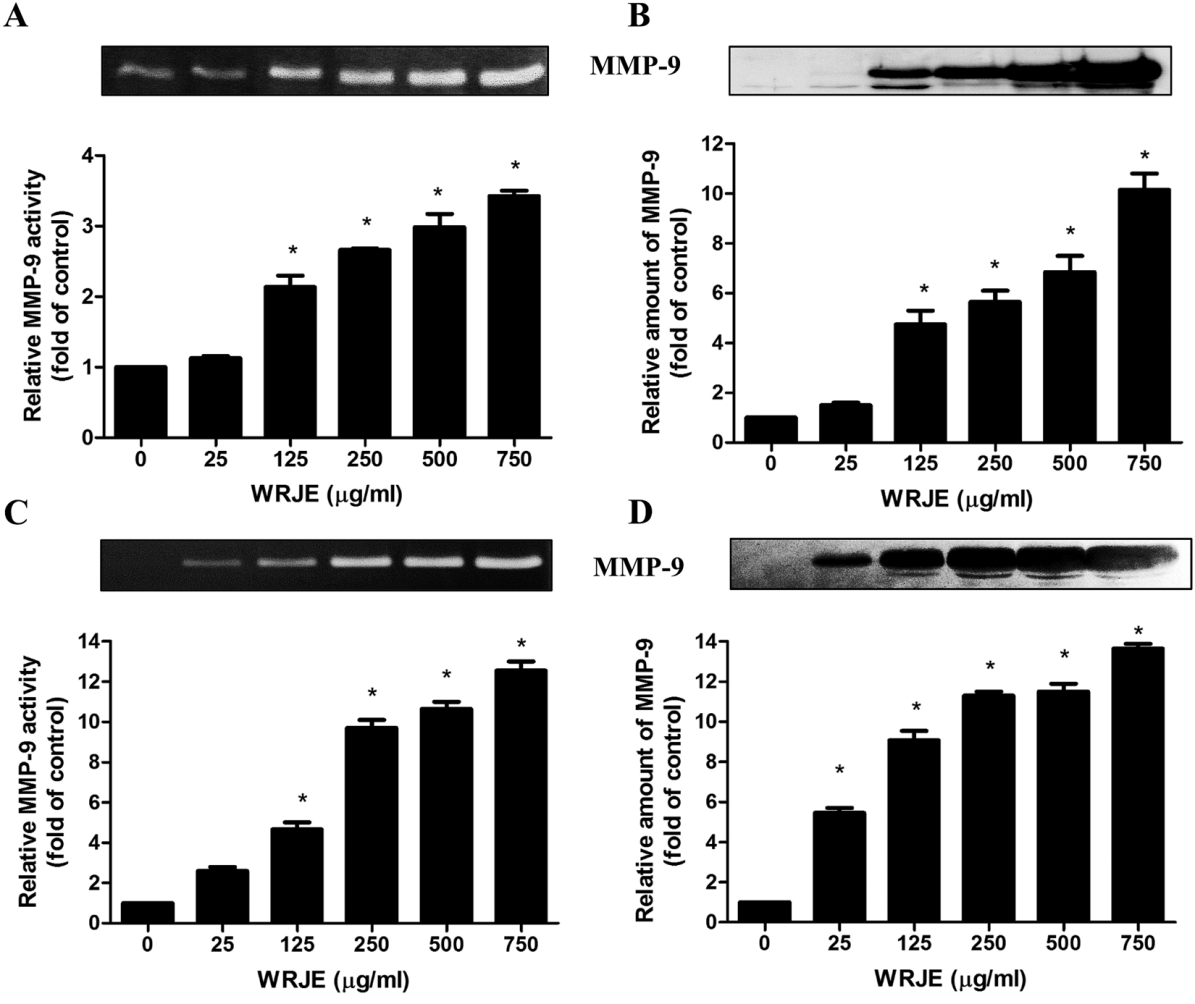
The effect of a water royal jelly extract (WRJE) on matrix metalloproteinase 9 (MMP-9) secretion and proteolytic activity in HaCaT cells (**A**,**B**) and human epidermal keratinocytes (HEK) (**C**,**D**). HaCaT and HEK cells were treated with different doses of WRJE for 72 h. (**A**,**C**) Conditioned equal volumes of the culture media were collected and subjected to gelatine zymography. Densitometric quantification of MMP-9 activity in culture media is presented. (**B**,**D**) Conditioned equal volumes of the concentrated culture media were subjected to 10% SDS-PAGE gels. MMP-9 (92 kDa) was detected by Western blotting and quantified densitometrically. The gels were run under the same experimental conditions. Shown are cropped gels/blots. (The gels/blots with indicated cropping lines are shown in Supplementary Fig. S2). Data are expressed as means and SEMs of three independent measurements. Asterisks indicate a significant difference from the untreated group, *P < 0.001.

To investigate the effect of WRJE on secreted MMP-9 in cultured medium, HaCaT and HEK cells were treated with the indicated concentrations of WRJE. The culture media were harvested 72 h after WRJE treatment to measure the relative activity and amount of MMP-9 by zymography and Western blot analysis, respectively. Relative MMP-9 activity and amount were significantly increased by WRJE treatment in a dose-dependent fashion. HEK cells were more sensitive to WRJE treatment than HaCaT cells regarding both activity and amount of secreted MMP-9 (Fig. 1A–D).

**Figure 2:**
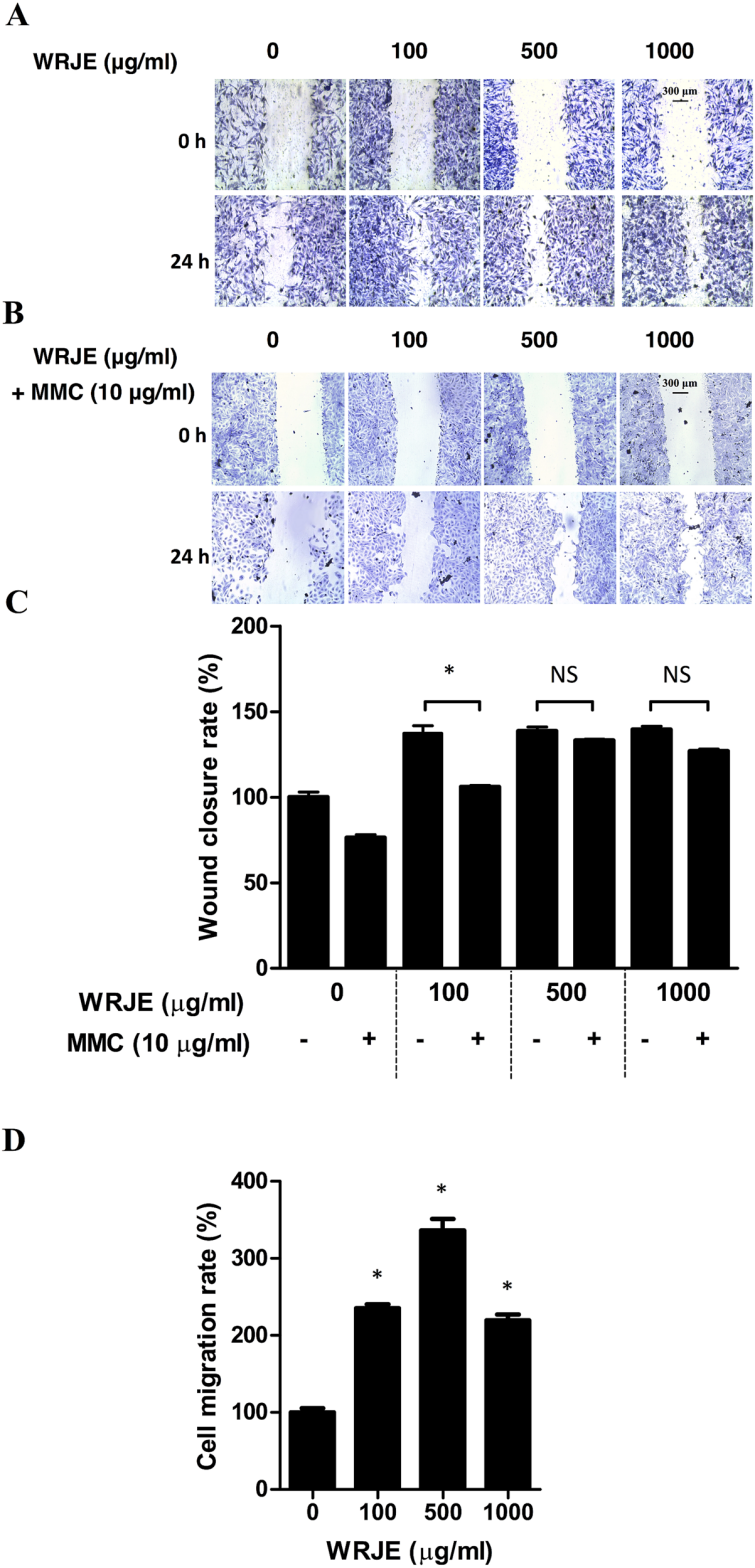
*In vitro* wound healing properties of a water royal jelly extract (WRJE) in HaCaT cells. Scratch wound analyses were performed in confluent monolayers of HaCaT cells. (**A**) Wounded cells were treated with different doses of WRJE for 24 h. (**B**) Scratch wounding with HaCaT cells was also conducted by pre-treating HaCaTcells with 10 μg/ml mitomycin C for 2 h and subsequent treatment with WRJE for 24 h. (**C**) Wound closure rates were determined as the difference between wound width at 0 and 24 h. (**D**) HaCaT cell migration was evaluated by the transwell assay plate (8 μm pore size, ThinCert™ 24 Well Cell Culture Inserts), where the cells were treated with different doses of WRJE for 24 h. Asterisks indicate a significant difference from the untreated group, *P < 0.001. Scale bar = 300 μm. NS: non-significant﻿.

Furthermore, we investigated the effect of WRJE at three concentrations (100, 500 and 1000 µg/ml) on mechanically scratched HaCaT cells cultured in 96-well plate. After a 24 h, scratched cells, incubated with WRJE exhibited significantly increased wound closure rates with respect to controls (*P* < 0.001) (Fig. 2A,C). In order to know whether the wound closure was influenced by an increased cell proliferation upon exposure to WRJE, wound healing assay was carried out in the presence of mitomycin C (MMC). As shown in Fig. 2B,C, MMC pre-treatment delayed re-epithelialisation of the wound area only at the lowest WRJE concentration (100 µg/ml), whereas no delay in re-epithelialisation was observed at higher WRJE concentrations within 24 h. Moreover, WRJE significantly increased the cell migration rates at all concentrations compared to with those of the controls, suggesting that WRJE possesses chemoattractant properties for keratinocytes (Fig. 2D). However, the observed effect was not dose-dependent suggesting that other WRJE components such as 10-HDA may counteract the cell migration activity.

### RJ contains MMP-9 inducer peptide, defensin-1

Before screening for the presence of MMP-9 inducer in lyophilised commercially available RJ from Yamada Bee Farm, we tested several Slovak RJ samples from different honeybee colonies and apiaries. All RJ samples increased MMP-9 activity; however, the observed enhancement was not uniform and suggests that MMP-9 inducer is a regular but quantitatively variable component of RJ (Supplementary Fig. S5).

**Figure 3:**
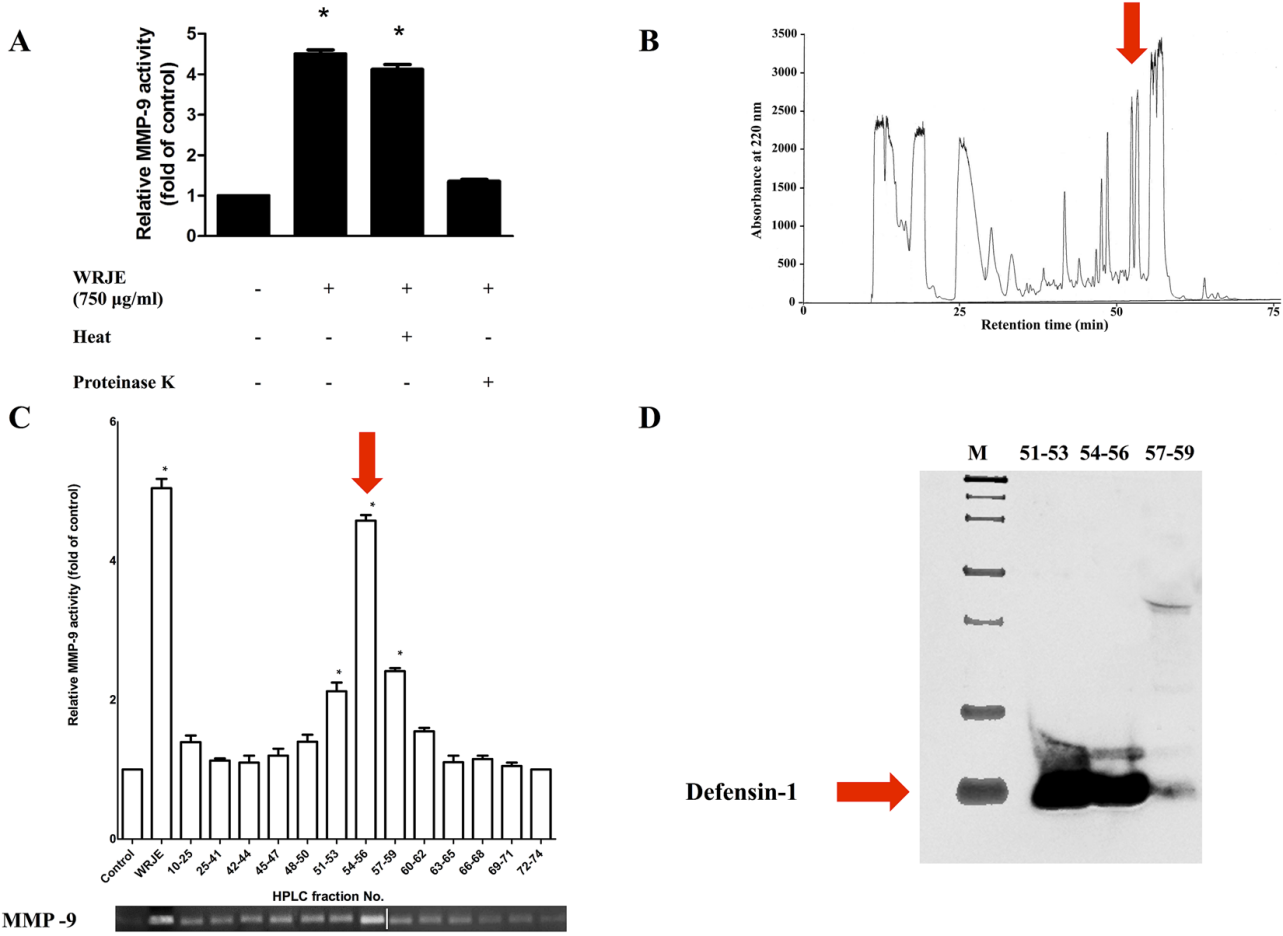
Characterisation and purification of the royal jelly (RJ) component responsible for elevated MMP-9 production. (**A**) Heat and proteinase K treatment was performed by incubation of water royal jelly extract (WRJE) at 100 °C for 5 min and incubation with 150 μg/ml proteinase K for 1 h at 40 °C followed by heating to 98 °C for 10 min to inactivate the enzyme. Treated WRJE was incubated with HaCaT cells and conditioned equal volumes of the culture media were collected and subjected to gelatine zymography. Densitometric quantification of MMP-9 activity in culture media is presented. (**B**) Heat-treated WRJE was fractionated by a reverse phase-high performance liquid chromatography (RP-HPLC) on a C18 column (250 × 4.6 mm, 5 μm) at a flow rate 0.3 ml/min, with elution using a 10–90% gradient of acetonitrile (containing 0.1% (v/v) trifluoroacetic acid) for 85 min. (**C**) The HPLC fractions were assayed for MMP-9 induction. (**D**) HPLC fractions with maximal MMP-9 activity (51 to 59 min) were used for identification of MMP-9 inducer and were subjected to 16.5% Tricine-SDS-PAGE gels. Defensin-1 (5.5 kDa) was detected by Western blotting using a rabbit polyclonal anti-honeybee defensin-1 antibody diluted 1:2000 in blocking buffer. Horseradish peroxidise-conjugated secondary antibodies were applied. (The gels/blots with indicated cropping lines are shown in Supplementary Fig. S3). White line in gel indicates the place where two gels were spliced together. Data are expressed as means and SEMs of three independent measurements. Asterisks indicate a significant difference from the untreated group, *P < 0.001.

Treatment of WRJE with proteinase K abrogated its MMP-9-induced activity, whereas heat-treatment had no effect, demonstrating that a heat-resistant proteinaceous WRJE component was sufficient for MMP-9 induction in this assay (Fig. 3A).

RP-HPLC chromatography was used to isolate the active peptide. WRJE was first heated and further fractioned on a C18 column (Fig. 3B). Activity was detected in a group of consecutive fractions eluted from the column at 51 to 59 min in the HPLC trace (Fig. 3C). Western blot analysis using a polyclonal antibody against Def-1 was further utilised to assess the fractions for the presence of Def-1, a heat-resistant bee peptide. Fractions displaying the highest activity were confirmed to be Def-1 based (Fig. 3D).

### Recombinant defensin-1 induces MMP-9 secretion and promotes re-epithelialisation *in vitro*

**Figure 4:**
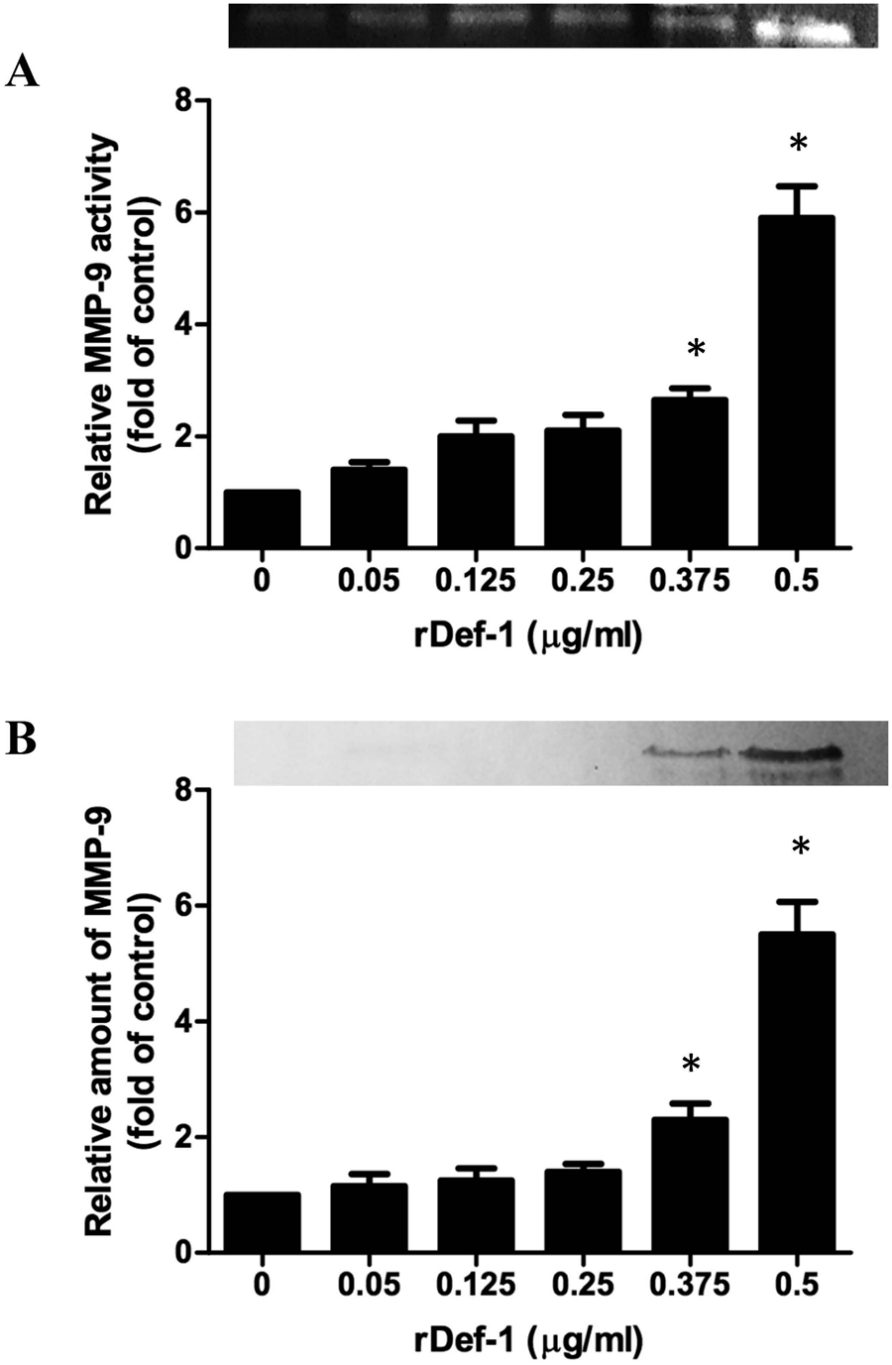
Induction of matrix metalloproteinase 9 (MMP-9) in HaCaT cells using recombinant defensin-1 (rDef-1). Relative amount of MMP-9 and its activity is presented following incubation of HaCaT cells for 72 h with rDef-1 at concentrations ranging from 0.05 to 0.5 µg/ml. (**A**) Conditioned equal volumes of the culture media were collected and subjected to gelatine zymography. Densitometric quantification of MMP-9 activity in culture media is presented. (**B**) Conditioned equal volumes of the concentrated culture media were subjected to 10% SDS-PAGE gels. MMP-9 (92 kDa) was detected by Western blotting and quantified densitometrically. The gels were run under the same experimental conditions. Shown are cropped gels/blots. (The gels/blots with indicated cropping lines are shown in Supplementary Fig. S4). Data are expressed as means and SEMs of three independent measurements. Asterisks indicate a significant difference from the untreated group, *P < 0.001.

To confirm that we identified a peptide with MMP-9 induction activity, we utilised the same assay previously used to screen WRJE by assessing the ability of rDef-1 to induce MMP-9 secretion from keratinocytes. To establish whether any effect was dose-dependent, we tested the effect of rDef-1 at a range of concentrations. As noted from the results of a representative experiment in Fig. 4, rDef-1 had a potent and dose-dependent effect on MMP-9 secretion from HaCaT cells. Recombinant Def-1 at a concentration of 0.375 µg/ml significantly enhanced the secretion of MMP-9.

**Figure 5:**
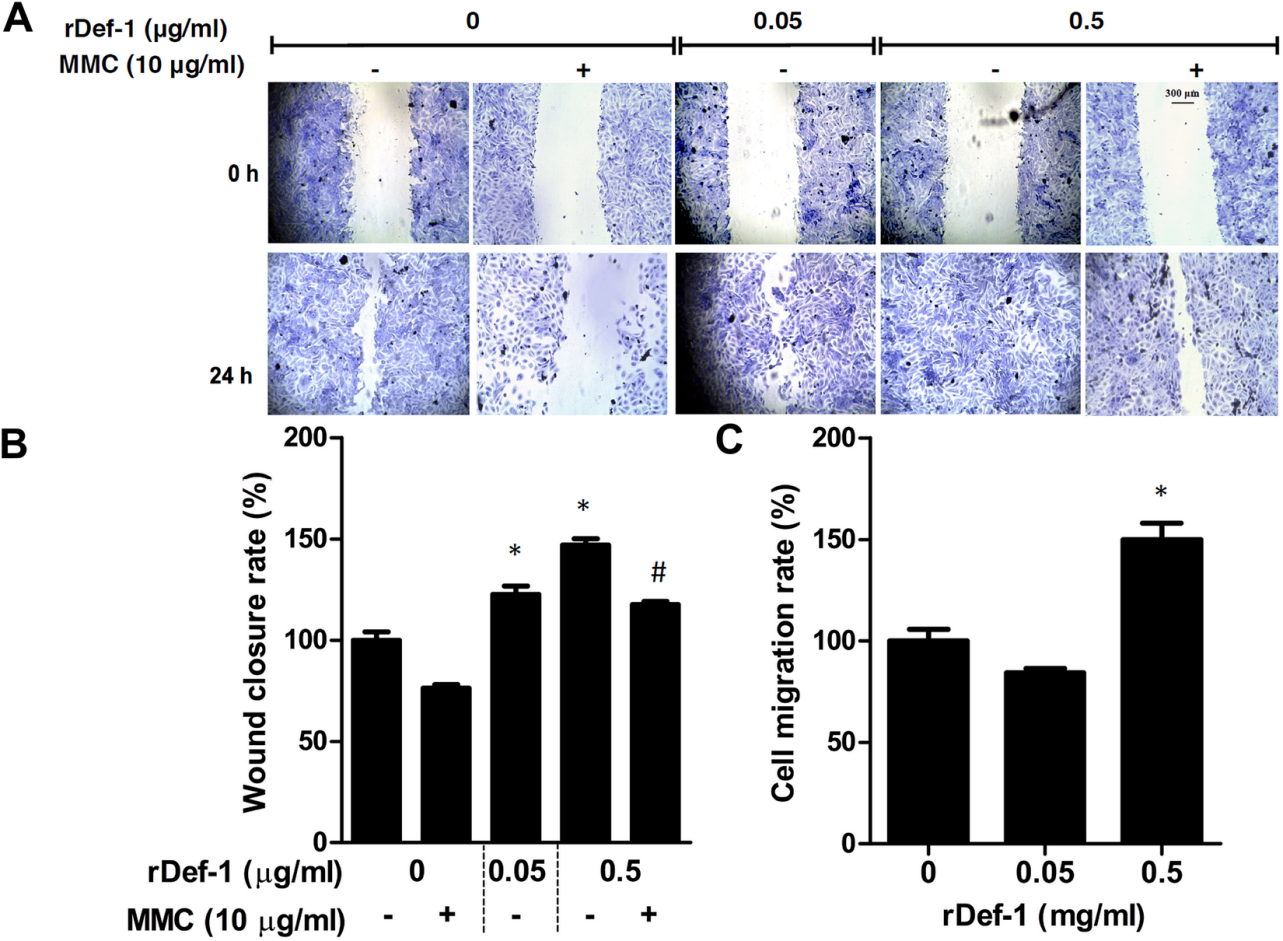
*In vitro* wound healing properties of a recombinant defensin-1 (rDef-1) in HaCaT cells. Scratch wound analysis was performed with confluent monolayers of HaCaT cells. (**A**,**B**) Wounded cells were treated either with rDef-1 at two concentrations (0.05 and 0.5 µg/ml) or with rDef-1 at 0.5 µg/ml after 2 h pre-treatment with mitomycin C, for 24 h. Wound closure rate was determined as the difference between wound width at 0 and 24 h. Symbol “#” indicate a significant difference from the rDef-1 group without mitomycin C, ^#^ *P* < 0.001. (**D**) HaCaT cell migration was evaluated by the transwell assay plate (8 μm pore size, ThinCert™ 24 Well Cell Culture Inserts), where the cells were treated with rDef-1 at two concentrations (0.05 and 0.5 µg/ml) for 24 h. Data are expressed as means and SEMs of three independent measurements. Asterisks indicate a significant difference from the untreated group, *P < 0.001. Scale bar = 300 μm.

To simulate wounding, a gap in a confluent monolayer of HaCaT cells was created with a pipette tip. In the presence of 0.05 and 0.5 µg/ml rDef-1, the rate of wound closure was significantly increased compared with the control at 24 h (Fig. 5A,B). Wound closure was almost complete or complete at 24 h in the presence of rDef-1, whereas it was incomplete in the control (Fig. 5A). Interestingly, wound healing activity of rDef-1 at concentration of 0.5 µg/ml was significantly suppressed by pre-treatment of HaCaT cells with MMC for 2 h. This suggests that proliferation of HaCaT cells highly contributes to the wound healing effect mediated by rDef-1. To obtain direct evidence regarding whether Def-1 affects cell migration, we used a chemotaxis assay. rDef-1 (0.5 µg/ml) significantly increased the number of migrating cells compared with controls (*P* < 0.001) (Fig. 5C) but the cell migration rate was lower than in case of WRJE.

To investigate the role of cell-signalling pathways in the effect of rDef-1 on wound closure, we performed scratch wound experiments using the ERK inhibitor PD98059 (10 µmol/l); p38-MAPK inhibitor SB203580 (20 µmol/l); cell-permeant calcium chelator BAPTA (30 µmol/l); and Ser/Thr protein kinase mTOR inhibitor Rapamycin (100 nmol/l). Confluent HaCaT cells were scratched in the absence or presence of each inhibitor with or without rDef-1, and wound closure was then measured 24 h post-wounding as described above. The greatest inhibitory effect (*P* < 0.001) was induced by PD98059, which totally abolished the wound repair effect of rDef-1 (Fig. S6). SB203580 and Rapamycin exerted a lower but statistically significant inhibition (*P* < 0.05) on rDef-1 compared with PD98059, whereas BAPTA exhibited no inhibition (Fig. S6).

### RJ and recombinant defensin-1 promotes re-epithelialisation *in vivo*

**Figure 6:**
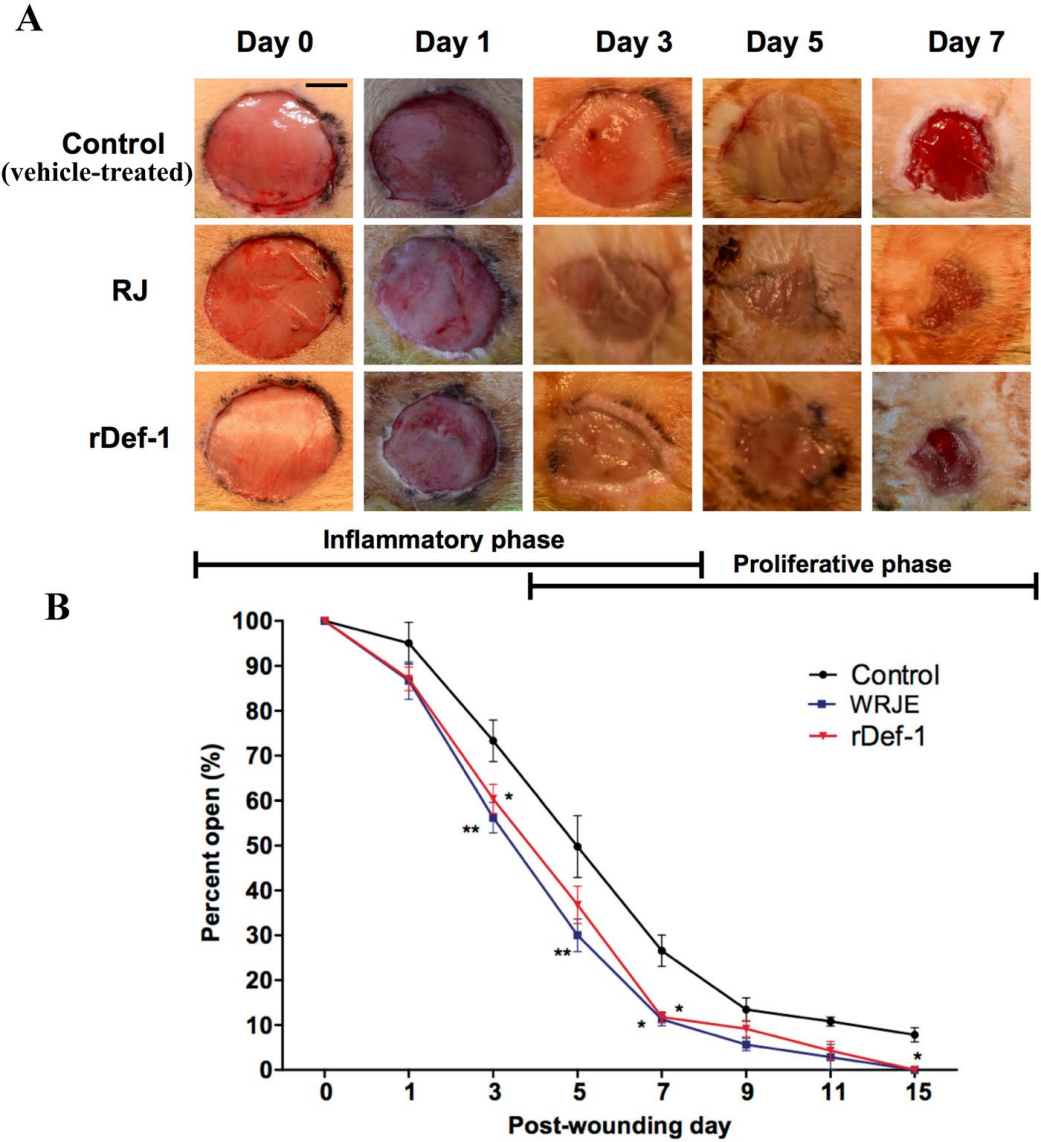
Typical macroscopic appearance of royal jelly (RJ) and recombinant defensin-1 (rDef-1) treated wounds compared with vehicle (carboxymethyl cellulose)-treated control wounds. RJ and rDef-1 treated wounds exhibited more rapid wound closure, especially at day 7 post-wounding (**A**). Wound healing curve (**B**) demonstrating significant differences between treated and control wounds. Data are expressed as means and SEMs of 4 to 20 independent measurements. Asterisks indicate a significant difference from the control (vehicle-treated) group, *P < 0.01, **P < 0.05. Scale bar = 5 mm.

**Figure 7:**
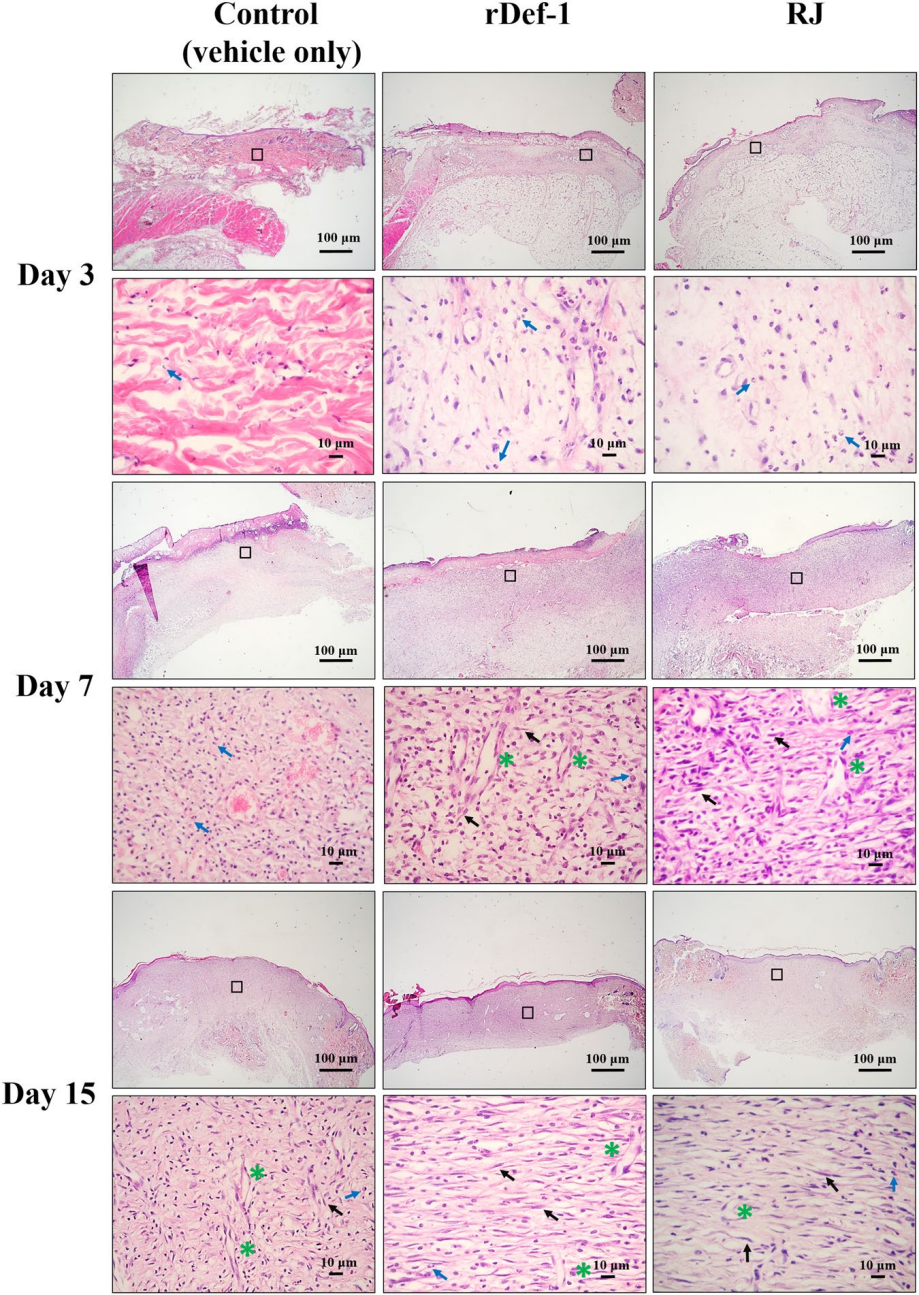
Representative pictures of haematoxylin and eosin-stained sections of wounds (n = 4 per time points) at days 3, 7 and 15 post-wounding exhibiting differences in epithelisation between vehicle (carboxymethyl cellulose)-treated and rDef-1- and RJ-treated wounds. Blue arrows indicate inflammatory cells. Black arrows indicate fibroblasts. Green asterisks indicate new blood vessels. Scale bar = 100 and 10 μm.

During the course of on-going wound healing, the areas of all wounds decreased, including untreated controls and RJ-treated and rDef-1-treated wounds, compared with the initial wound size. However, we observed significant differences in this parameter between the control (vehicle-treated only) and both treated wound groups (Fig. 6). RJ and rDef-1 markedly increased the wound closure, and complete epithelisation was observed on day 15 in these groups. Control wounds remained opened on day 15 (Figs 6 and 7). No significant differences between wound groups could be observed regarding the degree of granulation and vascularisation, but a strong trend for enhanced re-epithelisation was confirmed histologically with no differences between RJ and rDef-1 (Fig. 7). In the control vehicle-treated wounds at day 3 tissue detritus was observed on the surface of the wound that was transcended with decreased level of fibrin and sparse inflammatory infiltrate of neutrophils. The dermis is oedematous soaked with sparse infiltration of inflammatory cells. The neo-angiogenesis was not observed. At Day 7, ulceration was persisting and incipient formation of granulation tissue with angiogenesis together with the presence of neutrophils, macrophages and activated fibroblasts was observed. At Day 15, ulceration was still observed in a central part of the wound covering with tissue detritus and fibrin exudate and immigrant neutrophils and lymphocytes; however, re-epithelialization was started at the wound edge. In dermis, granulation tissue is present with vertically oriented capillaries, the activated fibroblasts and vertically and horizontally oriented bundles of collagen fibres (Fig. 7).

In the case of wound healing with RJ and rDef-1 we observed a similar histological finding (Fig. 7). At Day 3, the surface area of the wound was penetrated with a massive inflammatory infiltrate of neutrophils and fibrin exudate. The derma was oedematous soaked with stronger infiltration of inflammatory cells and activated fibroblasts with signs of angiogenesis. At day 7, re-epithelialization was observed at the wound surface with a mature epidermis with all layers. The granulation tissue is developed in the dermis with activated fibroblasts and neo-angiogenesis. At day 15 re-epithelialization of wound surface was progressed. Dermal oedema was declined and transformation of the granulation tissue into a scar with preserved dilated capillaries and predominantly horizontal oriented bundles of collagen fibres was observed.

Taken together, histological analyses showed that RJ as well as rDef-1 promoted a complete re-epithelialization of the wound surface and scar formation in the dermis in compare to vehicle (CMC) where only partial re-epithelialization was observed and the scar formation was missing.

## Discussion

In this work, WRJE and its Def-1 peptide promoted the secretion of MMP-9 by human keratinocytes in a dose-dependent manner, enhanced the migration of keratinocytes *in vitro*, and improved wound closure and re-epithelialisation in a rat excision wound model *in vivo*. Keratinocytes are the predominant cell type in the epidermis and are primarily responsible for the epithelialisation phase of wound healing. During epithelisation, keratinocytes loosen their cell-cell and cell-ECM contacts at the wound margin^21^. Keratinocytes proliferate and migrate from the proximal intact tissue to the wound site^22^. Early keratinocyte movement into the wound site is regulated by MMP-9^23^. MMP-9 is involved in processes that occur during cutaneous wound healing such as inflammation, matrix remodelling, and epithelialization. MMP-9 is required for the normal progression of wound closure^24^.

In our previous study, we demonstrated that honey significantly enhanced the expression of MMP-9 mRNA in primary keratinocyte cultures. Furthermore, incubation of human skin fragment with honey was associated with the increased expression of MMP-9, and the most robust staining was associated with epidermal keratinocytes. Honey also markedly promoted the degradation of collagen type IV, a substrate for MMP-9^25^. Interestingly, stimulation of MMP-9 expression was observed in honey samples of different botanical origins^26^.

One potential candidates that potentially participates in wound healing is MRJP1, which is the dominant honeybee protein present at high levels in both RJ and honey. Despite its numerous immunomodulatory and biological effects (reviewed in ref. 27), MRJP1 had very little or no effect on the expression and secretion of MMP-9 from human keratinocytes^25^. However, bee Def-1 significantly enhanced the secretion of MMP-9 from keratinocytes. Similar to MRJP1, Def-1 is a common but quantitatively variable factor present in both RJ and honey^8, 28^. The concentration of Def-1 in RJ and honey varied in amounts ranging from 0.159 to 0.524 µg/mg^8^ and 0.04 to 5.17 µg/g^28^, respectively.

Def-1 is an antibacterial peptide belonging to the insect defensin group that is composed of 51 amino acids, with a molecular weight of 5.52 kDa. Def-1 has a C-terminal extension of 12 amino acids with an α-helix structure^4^. Interestingly, bee and bumblebee defensins are the only C-terminal that is amidated, a feature often reported for cecropins^29^. Bee Def-1 is effective against Gram-positive bacteria^4, 30, 31^; however, some studies using recombinant Def-1 also reported its activity against Gram-negative bacteria including *Pseudomonas aeruginosa* and *Salmonella choleraesuis* ^32, 33^.

Although insect defensins were originally thought to be structurally similar to mammalian defensins, their three-dimensional structure and disulphide bridges patterns are different. Despite their structural differences, all defensins share a cationic character and exert antimicrobial activity presumably initiated by an interaction with the negatively charged membranes of pathogens^34^. In addition to their recognised antimicrobial activity, during the past decade, a plethora of studies have suggested that mammalian defensins are the cornerstone to the wound healing process. Human β-defensins are expressed at wound sites and were shown to stimulate various cellular activities, including keratinocyte proliferation, migration and wound healing.

To date, very few studies have investigated the immunomodulatory/wound healing properties of insect defensins^35, 36^. Recently, Lee and co-authors (2013) showed that treatment of *Staphylococcus aureus*-infected excision wounds with the defensin-like beetle peptide coprisin accelerated the wound healing rate by promoting reepithelisation and neovascularisation. Interestingly, coprisin incorporated into an ointment base (white petroleum) exerted greater effects compared with liquid base (saline solution). Wounds treated with coprisin contained more fibroblasts and leukocytes compared with wounds treated with saline solution. In this study, excision wounds treated with rDef-1 exhibited similar features with a higher infiltration of fibroblasts and keratinocytes into the wounds compared with vehicle-treated wounds. Another very promising insect defensins with immunomodulatory activities are lucifensins^35^. Lucifensins were identified in both larval tissues and maggot excretions/secretions of *Lucilia sericata* and *Lucilia cuprina*. Recently, an extract prepared from *L*. *sericata* maggots improved wound healing of rat wounds in terms of quicker wound closure rates and more rapid growth of keratinocytes and fibroblasts^37^. Although the authors elucidated the major molecular effects of maggot extract, which can accelerate wound healing through the enhanced activities of TGF-β/Smad3 and STAT3 signalling pathways, they did not identify maggot wound healing element(s) responsible for the observed effects.

To obtain more insight into WRJE and bee Def-1-stimulated wound closure, we performed a test using a MMC in order to investigate the contribution of cell proliferation in re-epithelialisation process *in vitro*. Interestingly, MMC delayed re-epithelialisation in rDef-1-treated keratinocytes but had no effect on re-epithelialisation process in WRJE-treated cells. This suggest that proliferation is crucial in Def-1-mediated wound healing process.

In addition, we also tested a battery of inhibitors of cell signal translation pathways that are involved in wound healing. Treatment with the ERK inhibitor PD98059 negatively affected the Def-1-induced wound closure rate. It has been shown elsewhere that PD98059 also inhibits MMP-9 induction and keratinocyte cell migration^38^. Hence, Def-1 may increase wound closure and cell migration due to the induction of MMP-9 secretion. Similarly, PD98059 was the strongest inhibitor of wound healing activity stimulated by different types of honey, suggesting that the observed honey wound healing activity was mediated by Def-1^26^. In addition, Tonks and colleagues identified a 5.8 kDa component of manuka honey that is responsible for cytokine induction in human monocytes and the mechanism via which this component stimulates innate immune cells^39^. Although the isolated unknown component was heat-unstable in boiled honey, we hypothesise that it could be Def-1. To the best of our knowledge, Def-1 interacts with various large honey proteins/glycoproteins and is subsequently captured in aggregates after heating. Heat treatment of WRJE also caused partial denaturation of Def-1, but the concentration of Def-1 in RJ is significantly increased compared with that in honey (data not shown).

As a key immunomodulatory component of RJ and honey, Def-1 may share some functional similarities with human β-defensins despite its distinct three-dimensional structure. The mechanism by which β-defensins exert their wound healing properties is not fully known. It has been suggested that hBD3, a highly expressed defensin in keratinocytes, promotes the proliferation and migration of keratinocytes through phosphorylation of epidermal growth factor receptor and STAT proteins as mentioned above^40^ and significantly accelerates wound closure when topically applied in a porcine model of infected skin wounds^41^.

β-defensins have a variety of different functions that are determined by the level of expression. Possibly, defensins combine pro- and anti-inflammatory effects depending on disease state and pathogen exposure^42^. Similarly, honey and its components either stimulate or inhibit the release of certain cytokines (tumour necrosis factor-α, interleukin-1β, interleukin-6) from human monocytes and macrophages, depending on wound conditions. Furthermore, honey seems to either reduce or activate the production of reactive oxygen species from neutrophils, also depending on the wound microenvironment^27^.

In conclusion, these data indisputably demonstrate that Def-1, a common but concentration variable factor present in honey and RJ, contributes to cutaneous wound closure. These findings support the use of bee Def-1 in skin regeneration.

## Materials and Methods

### Materials

Human HaCaT cells were purchased from Cell Lines Service (Eppelheim, Germany). Dulbecco’s modified Eagle’s medium (DMEM), 10% foetal calf serum (FCS), L-glutamine, antibiotics mixture (100 IU/ml penicillin and 100 μg/ml streptomycin) and trypsin-ethylenediaminetetraacetic acid (EDTA) were purchased from Biochrom AG (Germany). Human epidermal keratinocytes (HEK) cultured in EpiLife^®^ medium supplemented with human keratinocyte growth supplement were purchased from Life Technologies (UK). A lyophilised sample of RJ that was standardized to include minimum 3.85% (*E*)*-*10-hydroxy-2-decenoic acid was obtained from Yamada Bee Company, Inc. (Japan). FlashBAC GOLD expression system and pOET vector were purchased from Oxford Expression Technologies (UK). BlueSript plasmid was purchased from GenScript (Hong Kong) and restriction enzymes *XhoI* and *BamHI* from New England Biolabs (UK). *Spodoptera frugiperda* Sf9 cells obtained from Invitrogen (Germany) were grown in Sf-900II serum-free medium from Gibco (USA). Anti-MMP-9 antibody was purchased from Merck (Germany). Rabbit polyclonal anti-bee defensin-1 (Def-1) was purchased from GenCust Europe (Luxembourg), and horseradish peroxidise-conjugated secondary antibodies were obtained from Promega (USA). The following inhibitors were used in the HaCaT scratch wound assay: PD98059 (extracellular signal-regulated kinase [ERK] inhibitor, 10 μM), SB203580 (p38 inhibitor, 20 μM), BAPTA-AM (cell-permeant calcium chelator, 30 μM) and rapamycin (mammalian target of rapamycin [mTOR] inhibitor, 100 nM). These agents were obtained from Calbiochem (USA). All other reagents including mitomycin C were purchased from Sigma-Aldrich (Germany) unless otherwise stated.

### Cell culture

HaCaT keratinocytes were cultured in DMEM and sub-cultured every 4 days at 37 °C in 5% CO_2_. For all experiments, cells were grown to 70 to 80% confluence and incubated in serum-free DMEM 24 h prior to treatment with water RJ extract (WRJE) or recombinant Def-1 (rDef-1). Medium was then replaced with fresh serum-free DMEM, and cultures were treated with different concentrations of WRJE (0.25–1000 μg/ml) or rDef-1 (0.05–0.5 μg/ml) for 72 h.

HEK cells were sub-cultured according to the manufacturer’s instructions, and cultures were treated with WRJE or rDef-1 as mentioned above.

### Cell viability

The cytotoxic effect of WRJE on HaCaT cells or HEK was measured by the Alamar Blue assay (Life Technologies, UK) according to the manufacturer’s protocol. Results were expressed as the percentage of cytotoxicity calculated according to the manufacturer’s equation.

### Water royal jelly extract preparation

RJ was suspended in sterile deionised water at a concentration of 100 mg/ml. The supernatant of the WRJE was collected by centrifugation at 16,000 *g* for 30 min, divided into portions and stored at −80 °C until use. Total protein content in the WRJE was measured using the Quick Start Bradford protein assay (Bio-Rad, CA, USA) as described in the instruction manual.

### Heat and proteinase K treatment

Heat and proteinase K treatment were performed by incubation of WRJE at 100 °C for 5 min and WRJE with 150 μg/ml proteinase K for 1 h at 40 °C followed by heating to 98 °C for 10 min to inactivate the enzyme.

### WRJE fractionation

Heat-treated WRJE was fractionated by a reverse phase high performance liquid chromatography (RP-HPLC) on a C18 column (250 × 4.6 mm, 5 μm) at a flow rate of 0.3 ml/min with elution using a 10 to 90% gradient of acetonitrile (containing 0.1% (v/v) trifluoroacetic acid) for 85 min. HPLC fractions were freeze-dried under vacuum, re-dissolved in PBS, and assayed for MMP-9 induction. The fraction with maximal activity was used for identification of the MMP-9 inducer.

### Defensin-1 cloning, expression and purification

The cDNA fragment optimised for codon usage in *Spodoptera frugiperda* (Sf9) cells coding the signal peptide and mature bee defensin-1 followed by a (His)_6_-tag with *XhoI* site on the N-terminal and *BamHI* site on the C-terminal was synthesised and cloned into a BlueSript plasmid (GenScript, Hong Kong). The purchased BlueScript-Def plasmid was digested with *XhoI* and *BamHI*. The cDNA fragment was purified and then ligated into similarly digested pOET2 vector (Oxford Expression Technologies Ltd., UK). The resulting recombinant plasmid was transformed into JM 109 *Escherichia coli* and verified by DNA sequence analysis (GATC Biotech, Germany). The correct plasmid encoded a translational peptide containing an N-terminal signal peptide, followed by the mature peptide sequence of bee Def-1 and a (His)_6_-tag (hereafter designated rDef-1).

Recombinant baculovirus was obtained using the approach of Posse *et al*.^43^. Briefly, a Sf9 cell monolayer was co-transfected with flashback baculovirus (Oxford Expression Technologies, UK) and recombinant pOET2 transfer vector (described above), using Lipofectin (Thermo Fisher Scientific, MA, USA) following the manufacturer’s instructions. Recombinant virus was amplified by infection of Sf9 cells in serum-free Sf-900 II SFM culture at a low multiplicity-of-infection (moi), and the amplified virus was used to infect Sf9 liquid cultures at moi = 2 for protein expression. Viral titre was assessed by plaque assay. Then, 72 h after infection, culture medium was cleared by centrifugation (2,000 *g*, 5 min). Supernatants were loaded onto a SP Sepharose FF column (GE Healthcare, UK) equilibrated with 0.05 M phosphate buffer (pH 6.6, buffer A) and eluted using 0.5 M NaCl in buffer A.

The rDef-1 containing eluate fractions were pooled, adjusted with Triton X-100 to a final concentration of 0.1%, loaded onto 4 ml Ni Sepharose Excel resin (GE Healthcare), and eluted using 0.5 M imidazole. The peptide-containing eluate fractions were pooled again and desalted using desalting PD-10 columns (GE Healthcare). The purity of prepared rDef-1 in distilled water was determined by 16.5% Tricine-SDS-PAGE. The gel was stained with Serva Blue (Serva, Germany), and the concentration of rDef-1 was measured using the Quick Start Bradford Protein Assay (Bio-Rad).

### Gelatine zymography

Conditioned media of HaCaT and HEK cell cultures were subjected to gelatine zymography as previously described^44^. Briefly, non-reducing LDS sample buffer (Life Technologies, UK) was added to aliquots of culture medium supernatants at a ratio of 1:4. Twenty microlitre aliquots were separated on 8% SDS-PAGE gels containing 0.5 mg/ml gelatine under non-reducing conditions. The gels were washed in 2.5% Triton X-100 for 60 min at room temperature to remove the SDS and were subsequently incubated in a developer buffer [50 mM Tris (pH 7.8), 5 mM CaCl_2_ and 0.2 M NaCl] for 24 h at 37 °C. Gels were stained with 0.5% Coomassie Brilliant Blue G-250, and the bands of proteolytic activity were quantified by densitometry (Quantity One, Bio-Rad, USA).

### Western blot analysis

Western blot analysis was performed using the semi-dry blotting method^45^. Concentrated supernatants derived from HaCaT cells cultured as described above were prepared as follows. An initial volume of 0.5 ml of culture supernatant was collected from each well (control and treated cultures). A 10-fold concentration was obtained using ultracentrifugal filter devices (10,000 MWCO; Sartorius, Germany). Equal volumes (15 μl) of the concentrated supernatants were subjected to electrophoresis using 10% SDS-PAGE gels. Proteins were transferred onto nitrocellulose membranes and probed with the anti-MMP-9 antibody diluted at 1:400 in blocking buffer. Detection was performed using horseradish peroxidise-conjugated secondary antibodies. Visualisation of the immunoreactive bands was performed using the enhanced chemiluminescence kit (Kodak, USA). Quantification was performed by densitometry (Quantity One, Bio-Rad).

For detection of native Def-1 and its recombinant form (rDef-1), a rabbit polyclonal anti-bee Def-1 antibody was utilised according to Valachova *et al*. (2016). Briefly, fractions from Def-1 or rDef-1 purification were electrophoresed (15 μl) on a 16.5% Tricine-SDS-PAGE gel. Proteins were semi-dry blotted as mentioned above and probed with the rabbit polyclonal anti-honeybee Def-1-1 antibody diluted 1:2000 in blocking buffer. Detection was performed using horseradish peroxidise-conjugated secondary antibodies. Immunoreactive bands were detected using a solution containing dissolved SigmaFast 3,3-diaminobenzidine tablets (Sigma-Aldrich, UK).

### *In vitro* scratch wounding

Scratch wound analysis was performed on confluent HaCaT monolayers as described by Ranzato *et al*.^46^ with modifications. The width of the wound space was measured at wounding and at the end of treatment, via an inverted microscope equipped with a camera (Leica, Microsystem, Milan, Italy) and NIH ImageJ software (Bethesda, MD, USA). Wound closure was determined as the difference between wound width at 0 and 24 h. Briefly, HaCaT cells were seeded at 1 × 10^4^ cells/well in 96-well plates and grown to confluency in a complete DMEM medium. A linear wound was then generated in the monolayer with a sterile 200-μl plastic pipette tip. WRJE and rDef-1 were used at 100 to 1000 μg/ml and 0.05 to 0.5 μg/ml, respectively. Where indicated, the cells were treated with various inhibitors and 0.5 μg/ml rDef-1 for 24 h.

Scratch wounding with HaCaT cells was also conducted by pre-treating these cells with 10 μg/ml MMC for 2 h in order to assess the contribution of cell proliferation in the WRJE- and rDef-1-induced *in vitro* wound closure.

### *In vitro* cell migration assay

A cell migration assay was performed according to Ranzato *et al*.^26^ in transwell plates (8 μm pore size, ThinCert™ 24 Well Cell Culture Inserts for Multiwell Plates, Germany). Briefly, a total of 1 × 10^5^ cells per well were seeded in the upper compartment of filters. The lower chamber was filled with 500 μl complete DMEM medium with WRJE and rDef-1 at concentrations 100 to1000 μg/ml and 0.05 to 0.5 μg/ml, respectively. After 24 h incubation at 37 °C, the filters were removed, stained with 0.5% crystal violet (145 mM NaCl, 0.5% formal saline and 50% ethanol) for 10 min and washed thrice with water. The upper side of the filters was scraped using a cotton swab to remove cells that had attached but not migrated. Following PBS washing of the filters, the dye was eluted from the cells with 33% acetic acid and measured at 540 nm in a plate reader (Infinite 200 Pro, Tecan).

### *In vivo* wounding healing (excision model)

Adult male 10 to 13-week old Wistar albino rats (Velaz, Czech Republic) (n = 20) weighing 180–250 g were used. The experimental animals were housed separately and fed standard pelleted food with no restricted access to water and food during the course of experiment. The animals were anaesthetised with ketamine (Narkamon, Bioveta, Czech Republic) and xylazine (Cylariem, Germany) anaesthesia with inhalation introduction using isoflurane (Aerrane, Baxter, UK). After each painful intervention, the animals were supplied with tramadolum i.m. (Tramal, Grunenthal, Germany). Before the surgery, both flanks were shaved and disinfected with 70% alcohol. Four full thickness round excision wounds (1.5 cm in diameter) were induced (two on each side of the animal’s back). One wound was a untreated wound. The second wound was treated with vehicle only, namely carboxymethyl cellulose (CMC) gel. The third wound was treated with 5% RJ ointment in CMC. The fourth wound was treated with 0.1 mg/ml rDef-1 ointment in CMC. Wounds were dressed with Tegaderm sterile dressing (3 M Healthcare, MN, USA), which was changed every other day until wound closure. Digital photographs were taken at the time of surgery, and every other day until closure, which was defined as the time at which the wound was completely re-epithelialised and filled with new tissue. The diameter of the open wound area was measured. The wound closure area of each animal was assessed by tracing the wound diameter at days 0, 1, 3, 5, 7, 9 and 13 after wounding surgery and the wound closure rate was expressed as the percentage of open wound area.

### Histological analyses

Full profile biopsies, including central wound zone and adjacent unwounded skin, were obtained on days 1, 3, 7, 11 and 15 (four animals per time interval) and fixed in 4% paraformaldehyde. Serial paraffin sections (4 to 5 µm thick) were cut and stained with haematoxylin/eosin (HE) for blinded general morphological description and an assessment of wound healing parameters and pathology, such as the presence of necrosis, inflammatory reaction, granulation, angiogenesis, epithelisation and general granulation tissue morphology. Microscopic images were captured (microscope Opton AxioPhot, Germany) with the Zeiss Axio Vision imaging software.

### Study approval

All methods were performed in accordance with the national guidelines and regulations. The preparation of genetically modified *Escherichia coli* and recombinant peptide followed the guidelines of the Ministry of Environment of the Slovak Republic. All animal work was approved by the State Veterinary and Food Administration of the Slovak Republic (Project License 3743/14-221) following local ethical approval and was conducted in accordance with the Animal Care guidelines of the Slovak Medical University.

### Statistical analysis

Data were collected from three and at least four independent *in vitro* and *in vivo* experiments, respectively. The results are presented as the means ± standard errors (SEM). All data were statistically analysed using t-tests or one-way ANOVAs for comparisons of two groups or groups greater than three, respectively. *P*-values less than 0.05 were considered to be significant. Analyses were performed using GraphPad Prism (GraphPad Software Inc., CA, USA).

## Electronic supplementary material

Supplementary information
