# Supplementary material for: Bee-derived antibacterial peptide, defensin-1, promotes wound re-epithelialisation in vitro and in vivo
Source: Sci Rep. 2017 Aug 4;7:7340. doi: 10.1038/s41598-017-07494-0 (PMC5544694; doi:10.1038/s41598-017-07494-0)

Bee-derived antibacterial peptide, defensin-1, promotes wound re-epithelialisation in vitro and in vivo

Journal: Scientific Reports

Marcela Bucekova, Martin Sojka, Ivana Valachova, Simona Martinotti, Elia Ranzato, Zoltan Szep, Viktor Majtan, Jaroslav Klaudiny, and Juraj Majtan

Correspondence should be addressed to Dr. Juraj Majtan, Institute of Molecular Biology, Slovak Academy of Sciences, Dubravska cesta 21, 845 51 Bratislava, Slovakia. Phone: +421-2-59307438, Fax: +421-2-59302646, E-mail: juraj.majtan@savba.sk

Supplementary Fig. 1

Effects of WRJE on HaCaT cell viability using the Alamar blue assay. Cells were treated with the indicated concentrations of WRJE, and cell viability was determined by the Alamar Blue assay. Data are expressed as % of control and each column represents the mean ± SEM of independent four tests.

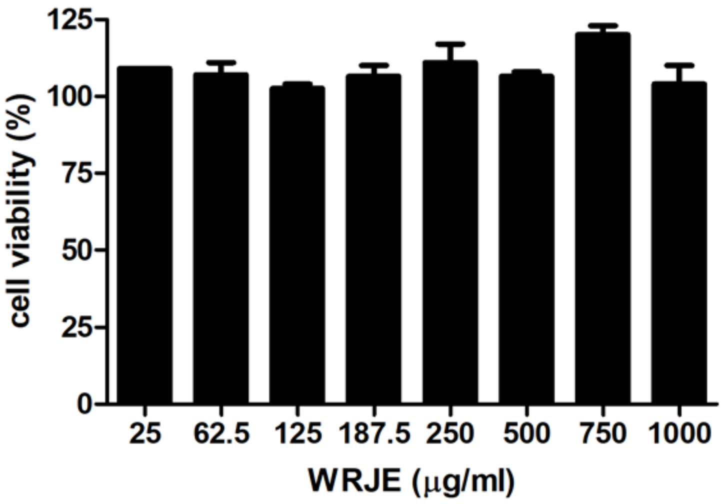

Supplementary Fig. 2

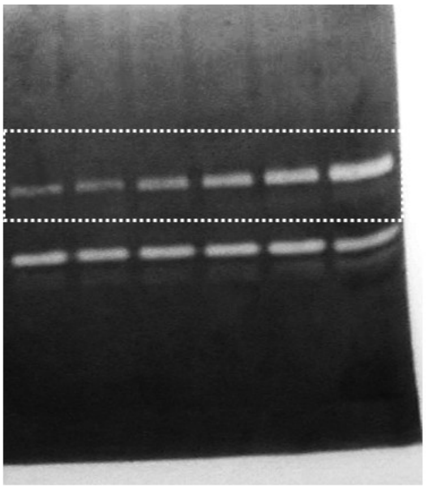

Fig. 1A

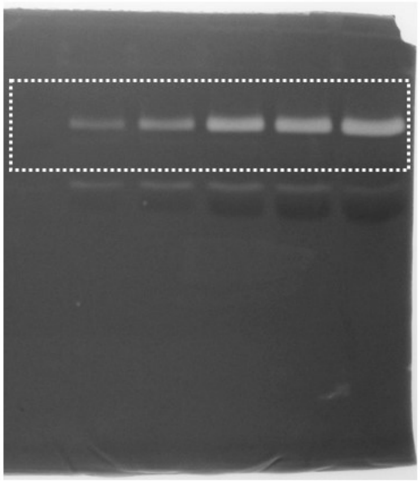

Fig. 1C

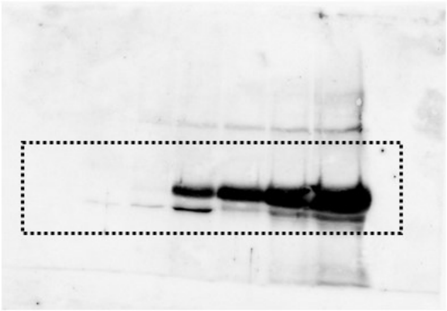

Fig. 1B

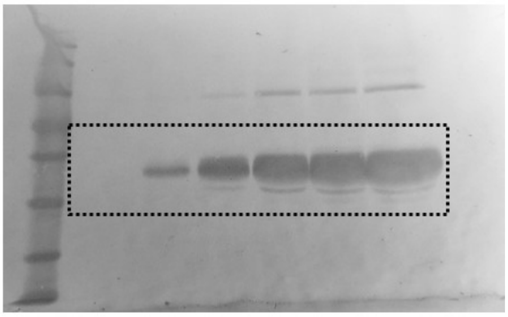

Fig. 1D

Supplementary Fig. 3

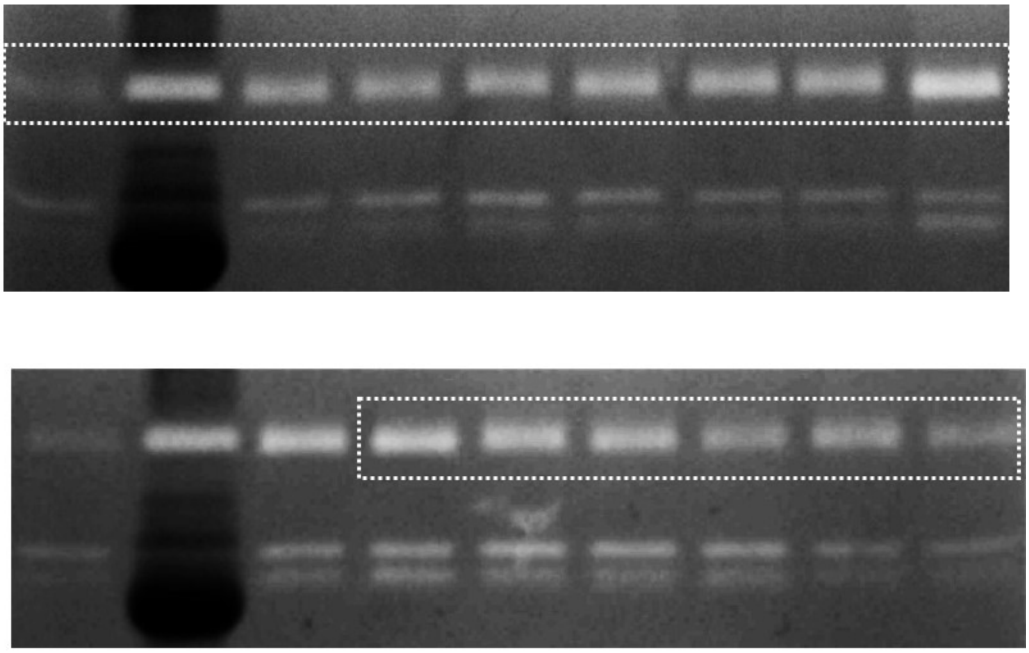

Fig. 3C

Supplementary Fig. 4

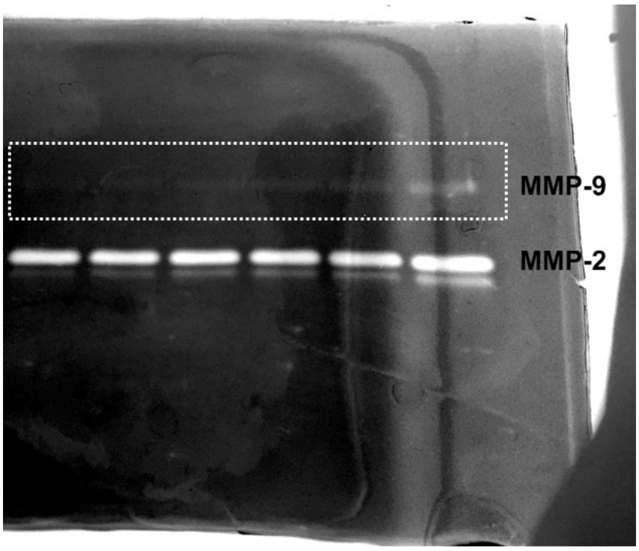

Fig. 4A

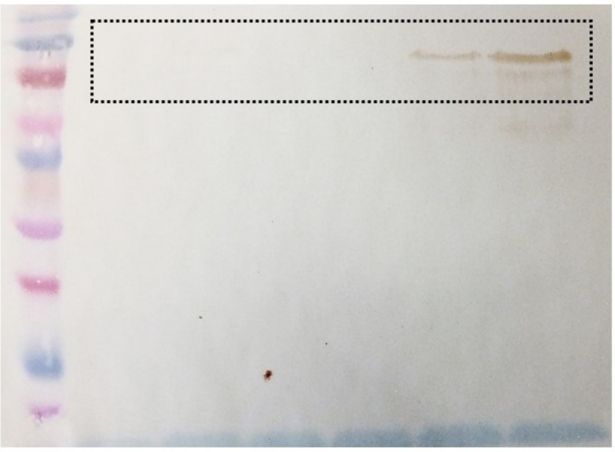

Fig. 4B

Supplementary Fig. 5

Effect of royal jelly (RJ) samples harvested from different apiaries on MMP-9 induction in HaCaT cells. HaCaT cells were treated with different doses of WRJE samples for 72 h. Conditioned equal volumes of the culture media were collected and subjected to gelatine zymography. Densitometric quantification of MMP-9 activity in culture media is present. Data are expressed as a mean with SEM of three independent measurements.

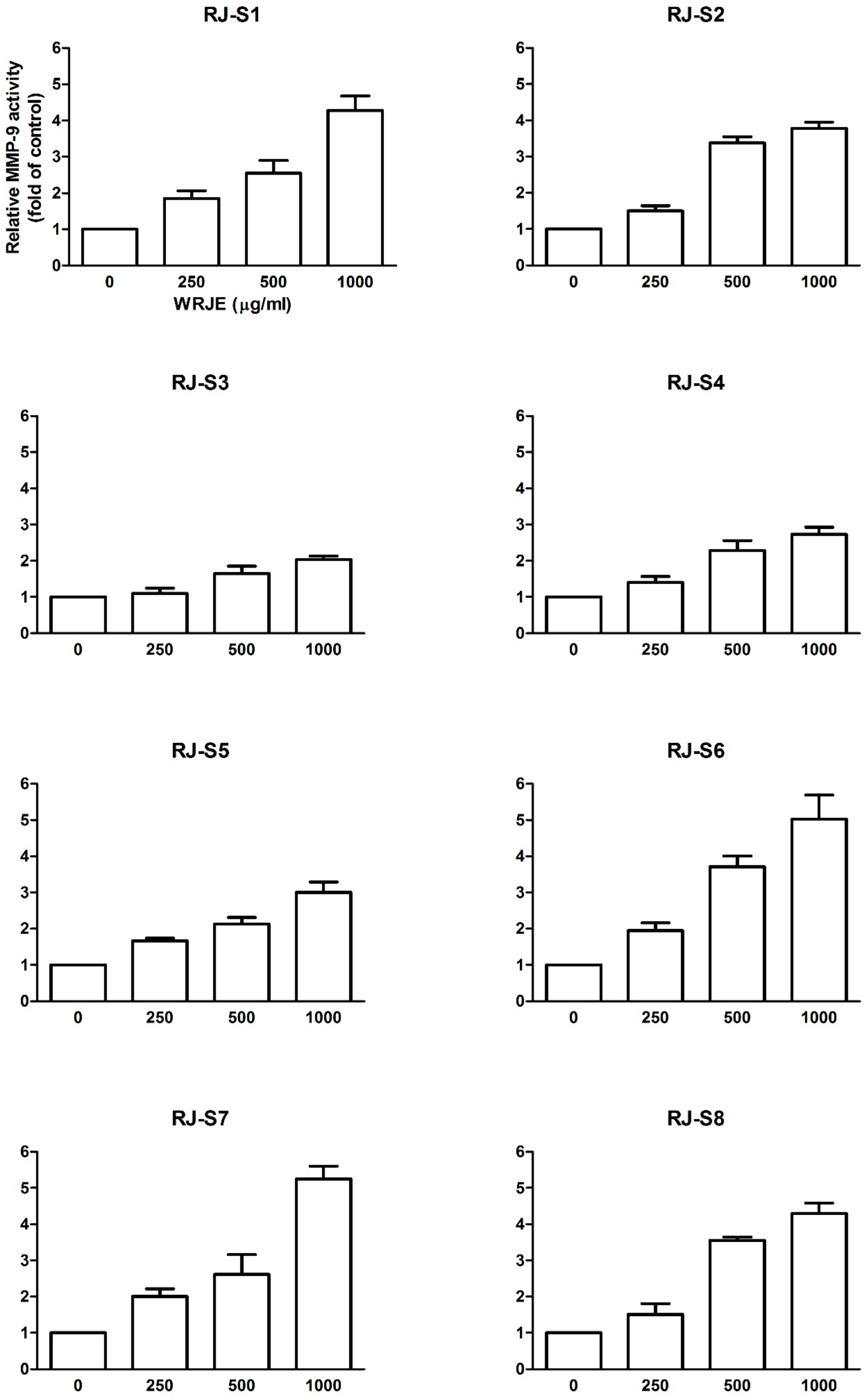
RJ-S1
RJ-S2
RJ-S3
RJ-S4
RJ-S5
RJ-S6
RJ-S7
RJ-S8
Relative MMP-9 activity
(fold of control)
WRJE (μg/ml)
0
250
500
1000
0
1
2
3
4
5
6

Supplementary Fig. 6

In vitro wound healing properties of a recombinant defensin-1 (rDef-1) in HaCaT cells. Scratch wound analysis was performed with confluent monolayers of HaCaT cells. Confluent HaCaT cells were scratched in the absence or presence of each inhibitor with or without 0.5 μg/ml rDef-1, and the wound closure was then measured 24 h post-wounding. Asterisk indicates a significant difference from Def-1-tretated group, $^{*}P<0.001$. Data are expressed as means and SEMs of three independent measurements.

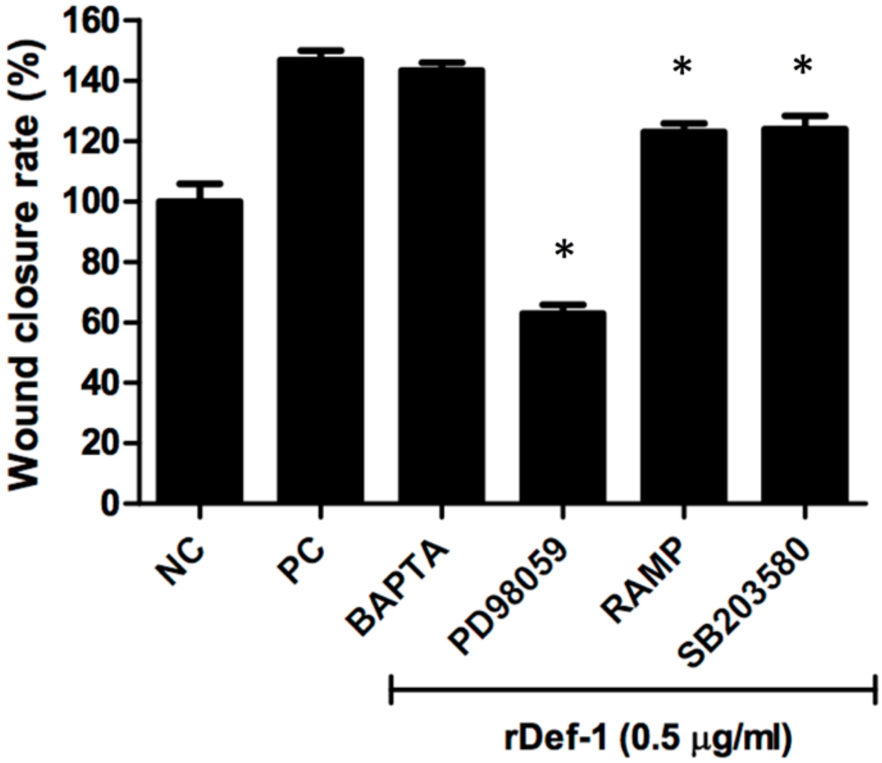

Supplement: Supplementary file 1 — Supplementary information [file 41598_2017_7494_MOESM1_ESM.pdf]
